# Activation of the spinal and brainstem locomotor networks during free treadmill stepping in rats lacking dopamine transporter

**DOI:** 10.3389/fnmol.2023.1299297

**Published:** 2023-11-21

**Authors:** Aleksandr Veshchitskii, Polina Shkorbatova, Aleksandr Mikhalkin, Zoja Fesenko, Evgeniya V. Efimova, Raul R. Gainetdinov, Natalia Merkulyeva

**Affiliations:** ^1^Neuromorphology Laboratory, Pavlov Institute of Physiology, Russian Academy of Sciences, Saint Petersburg, Russia; ^2^Institute of Translational Biomedicine, Saint Petersburg State University, Saint Petersburg, Russia; ^3^Saint Petersburg University Hospital, Saint Petersburg State University, Saint Petersburg, Russia

**Keywords:** dopamine transporter, treadmill locomotion, *c-fos*, spinal cord, mesencephalic locomotor area, ventrolateral periaqueductal gray

## Abstract

Dopamine is extremely important for the multiple functions of the brain and spinal cord including locomotor behavior. Extracellular dopamine levels are controlled by the membrane dopamine transporter (DAT), and animals lacking DAT (DAT-KO) are characterized by hyperdopaminergia and several alterations of locomotion including hyperactivity. Neuronal mechanisms of such altered locomotor behavior are still not fully understood. We believe that in hyperdopaminergic animals both the spinal and brain neuronal networks involved in locomotion are modified. Using the *c-fos* technique, we studied activated neuronal networks of the spinal cord and two brainstem structures related to locomotor control and being under the strong dopaminergic influence, the cuneiform nucleus (CnF) and ventrolateral periaqueductal gray (VLPAG), in wild-type (DAT-WT) and DAT-KO rats. In the spinal cord, most *c-fos*-positive cells were located in the dorsal laminae II-IV and in the central gray matter (laminae V-VI). No differences were revealed for the central areas. As for the dorsal areas, in the DAT-WT group, labeled cells mostly occupied the lateral region, whereas, in the DAT-KO group, *c-fos*-positive cells were observed in both medial and lateral regions in some animals or in the medial regions in some animals. In the brainstem of the DAT-WT group, approximately the same number of labeled cells were found in the CnF and VLPAG, but in the DAT-KO group, the VLPAG contained a significantly smaller number of *c-fos*-positive cells compared to the CnF. Thereby, our work indicates an imbalance in the sensorimotor networks located within the dorsal horns of the spinal cord as well as a disbalance in the activity of brainstem networks in the DAT-deficient animals.

## 1 Introduction

Dopamine is a neurotransmitter with multiple vital functions in both the brain (Centonze et al., 2001; Calabresi et al., 2007) and the spinal cord (Han et al., 2007; Sharples et al., 2014). Dopamine function is dependent upon its extracellular concentration, which is regulated by the plasma membrane dopamine transporter (DAT) (Giros et al., 1996). Rats and mice lacking a dopamine transporter (DAT-knockout, DAT-KO) have persistent hyperdopaminergia and are characterized by multiple alterations of cognitive and locomotor behaviors (Efimova et al., 2016; Reinwald et al., 2022). Some neuropsychiatric diseases like schizophrenia, attention deficit hyperactivity disorder, and bipolar disorder are also related to hyperdopaminergia (Rao et al., 1993; Maher et al., 2002; Cousins et al., 2009).

Despite most investigations being concentrated on the brain networks, the data about DAT-KO-related disturbances of locomotion like prominent hyperactivity and thigmotaxis (Giros et al., 1996; Rossi and Yin, 2015; Leo et al., 2018; Reinwald et al., 2022) point to possible alterations also in the spinal mechanisms of locomotor control. The spinal cord is one of the main nervous structures responsible for locomotion. The key locomotor networks are the central pattern generators (CPGs) located within the cervical and lumbar regions of the spinal cord (Arshavsky et al., 1997; Duysens and Van de Crommert, 1998; Yamaguchi, 2004; Grillner and Kozlov, 2021). CPG activity is under the supraspinal modulatory influence. The main modulatory supraspinal systems are monoaminergic, and dopamine is one of the monoamines with a modulatory influence on the spinal locomotor networks (Barrière et al., 2004; Clemens and Hochman, 2004; Humphreys and Whelan, 2012; Sharples et al., 2014). The main functions of the dopaminergic projections to the spinal cord are modulation of sensory processing, pain, motor, and visceral control (Lindvall et al., 1983; Fleetwood-Walker et al., 1988; Weil-Fugazza and Godefroy, 1993; Barrière et al., 2004). It might be expected that spinal mechanisms of locomotor control are altered in DAT-KO animals.

One of the ways to visualize the spinal locomotor networks is the *c-fos* technique, which allows us to recognize neuronal populations activated during motor tasks. This technique was repeatedly used in mammals like rats (Ahn et al., 2006) and cats (Dai et al., 2005; Noga et al., 2009; Merkulyeva et al., 2018). One of the drivers of spinal locomotor networks is the mesencephalic locomotor area (MLR), which is composed of the brainstem cuneiform nucleus (CnF) and pedunculopontine nucleus (Grillner and El Manira, 2020). Dopaminergic innervation of the MLR was shown in some vertebrates including mammals (Rolland et al., 2009; Ryczko et al., 2016; Sharma et al., 2018), and thereby an alteration in the MLR function might also be expected in DAT-KO animals. Recent data obtained from the systematic analysis of the afferent and efferent projections of the CnF and the behavioral responses to its optogenetic activation/pharmacological inactivation led to the assumption that this nucleus can be a caudal component of the periaqueductal gray (PAG) (Bindi et al., 2023). The PAG, mainly its ventrolateral region, modulates motor behavior (Morgan et al., 1998; Morgan and Carrive, 2001), and the PAG contains a dopaminergic population considered to be an extension of the dopaminergic A10 group (Dougalis et al., 2012). Both the brainstem structures CnF and PAG have increased amounts of the *c-fos*-labeled (FOS+) cells after locomotor activity (Brudzynski and Wang, 1996; Jordan, 1998). Therefore, in the present paper, we compared patterns for FOS+ cell distribution within the spinal cord and brainstem of DAT-KO and wild-type rats after treadmill stepping.

## 2 Materials and methods

### 2.1 Animals

Care and experimental procedures were carried out in accordance with requirements of Council Directive 2010/63EU of the European Parliament on the protection of animals used for experimental and other scientific purposes and the guidelines of the National Institute of Health Guide for the Care and Use of Laboratory Animals and with the approval of the Ethics Commission of the Saint Petersburg State University (Protocol No 131-03-06).

Adult Wistar male rats of wild-type littermates (DAT-WT, *n* = 7) and dopamine transporter knockout strain (DAT-KO, *n* = 7) aged 6.5–7.5 months were used. DAT-WT animals weighed 225–480 g, and DAT-KO had a lower weight: 200–285 g, as was previously documented (Leo et al., 2018; Apryatin et al., 2019; Mallien et al., 2022). A detailed description of the strain was previously reported in the paper (Leo et al., 2018). Animals were obtained from Saint Petersburg State University animal facility, housed two to three per cage, and maintained under standard lab conditions (12 h light/dark cycle) with food and water provided *ad libitum*.

### 2.2 Experimental design

**FIGURE 1:**
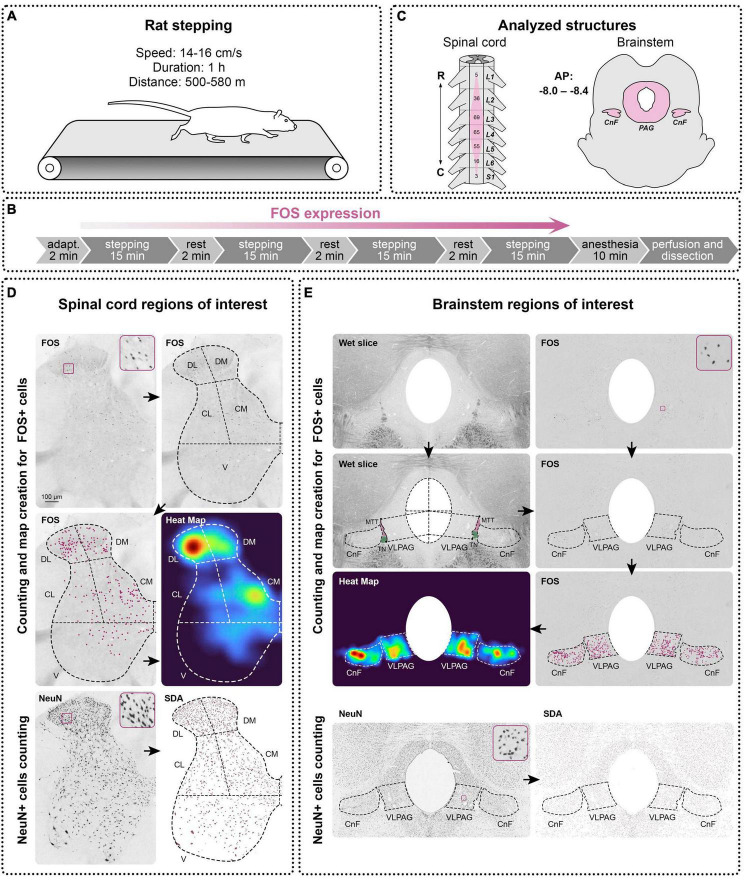
Design of the study. **(A)** A treadmill setting for rats stepping. **(B)** Experimental protocol for *c-fos* (FOS) expression. **(C)** Analyzed structures of the spinal cord and brainstem. R—rostral, C—caudal, A—anterior, P—posterior, L, S—lumbar and sacral segments of the spinal cord, CnF—cuneiform nucleus, PAG—periaqueductal gray, MTT—mesencephalic trigeminal tract, TN—trochlear nerve. **(D,E)** FOS and NeuN expression in the spinal cord and brainstem and methods of definition and analyzing of the regions of interest in them. DL, DM, CL, CM, V—dorsolateral, dorsomedial, centrolateral, centromedial, and ventral regions, VLPAG—ventrolateral periaqueductal gray. Heat Maps were created on the basis of manually defined FOS+ cells using the kernel density estimation algorithm, a statistical method for spatial point pattern visualization, which makes it more convenient to perceive the regional differences in the FOS distribution. Automatic method for object segmentation based on the statistical dominance algorithm (SDA) was used for counting of the NeuN-positive neurons in the spinal cord and brainstem. Enlarged images of examples of FOS-positive and NeuN-positive cells are presented in squares.

For visualization in the spinal cord and brainstem of neuronal networks active during locomotion, the expression of immediate early gene *c-fos* was immunohistochemically detected. For this aim, rats were stepped quadrupedally on a treadmill (Columbus, Exer-6M; USA) (Figure 1A). The duration of stepping was 60 min (Figure 1B) with an average speed of 14 ± 0.45 cm/s for DAT-WT rats and 16 ± 0.16 cm/s for DAT-KO rats. Under these conditions, rats of both groups were motivated to step but did not have vigorous fatigue; this speed was correspondent to speed diapason in the paper by Ahn et al. (2006). No electrical stimulation triggering stepping was used, and only light touching to the skin was performed. Short (2 min) rest intervals were used every 15 min; the total duration of the rest was 9–10% of the stepping duration. One non-stepped rat was also used for the *c-fos* protocol, as a control for basal locomotor activity.

### 2.3 Perfusion

Immediately after treadmill walking, animals were deeply anesthetized with a mixture of Zoletil (Virbac, France; 20 mg/kg) and Xyla (Interchemie werken “De Adelaar” BV, Netherlands; 2 mg/kg; i/p), and 10 min before the start of perfusion, heparin (Endopharm, Russia; 1 ml/kg) was injected intramuscularly. Thereafter, animals were transcardially perfused with 0.9% NaCl (200 ml, with Heparin, 0.5 ml/l) followed by 4% paraformaldehyde (350 ml). After perfusion, brains and spinal cords with dorsal root ganglia were removed from the cranium and spine, respectively, and stored consistently in 20 and 30% sucrose until they sank. For the spinal cords, the distances between the caudal-most parts of the dorsal rootlet attachment zones, connected to the neighboring dorsal root ganglia, were measured as spinal cord segments (Shkorbatova et al., 2019). The lumbar enlargement of the spinal cords was divided into segments. To assess the general anatomy of the lumbar spinal cord, lengths of the spinal segments (L1-L2, L3-L4, and L5-L6) were measured. Thereafter, brain and spinal cord segments were cut into 50 μm frontal and transverse slices, respectively, on a freezing microtome (Reichert, Austria). Slices were stored in 0.01 M phosphate-buffered saline (PBS) with 0.1% NaN_3_ at +4°C.

### 2.4 Immunohistochemical staining

Slices were processed as free floating. Between all procedures, the slices were washed in 0.01M PBS. Endogenous peroxidase activity was blocked by 0.3% H_2_O_2_, unspecific immunoreactivity was lowered by incubation in 3% bovine serum albumin (BSA, Biolot). Thereafter, slices were incubated for 70 h in primary antibody (Table 1) with 0.1% NaN_3_. Then, the slices were incubated for 24 h in a biotinylated secondary antibody (Table 1) with 1% BSA and 0.1% NaN_3_. Slices were then subsequently processed in an avidin-biotin horseradish-peroxidase complex (ABC Elite system, Vector Laboratories) and diaminobenzidine-NiCl_2_ solution. After washing in distilled H_2_O, slices were mounted, dehydrated, cleared, and placed under coverslips in Bio Mount HM (Bio-Optica Milano, Italy).

**TABLE 1:** The antibodies list.

	Antibody	Host	Clonality	Dilution	Manufacture	Lot	RRID
I	*c-fos*	Rabbit	Polyclonal	1:4,000	Sigma-Aldrich	ABE457	AB_2631318
	NeuN	Mouse	Monoclonal	1:5,000	Sigma-Aldrich	MAB377	AB_2298772
II	Anti-rabbit IgG	Sheep		1:600	Vector Lab	BA-1000	AB_2313606
	Anti-mouse IgG	Horse		1:600	Vector Lab	BA-2000	AB_2313581

I, primary antibodies; II, secondary antibodies.

### 2.5 Antibody characterization

All antibodies used (Table 1) were previously approved for use with rat tissue. Anti-*c-fos* antibody (Sigma-Aldrich, ABE457, RRID:AB_2631318) is a rabbit polyclonal IgG recognizing 56 kDa bands on Western blots of a rat nervous tissue (Javed et al., 2022). The immunohistochemical staining of the rat brain and spinal cord slices (used at 1:4,000) produced a pattern of FOS+ labeling that corresponds with the results of the previous studies (Jordan, 1998; Ahn et al., 2006), the population of neurons activated during stepping. Anti-NeuN antibody (Sigma-Aldrich, Cat.# MAB377, RRID:AB_2298772) is a mouse monoclonal IgG1 recognizing 46 and 48 kDa bands on Western blots of a rat nervous tissue (Darlington et al., 2008). The immunohistochemical staining of the rat brain and spinal cord slices (used at 1:5,000) produced a pattern of NeuN labeling that corresponds with the results of the previous studies (Loyd et al., 2008; Watson et al., 2009), the total population of exclusively neuronal cells.

### 2.6 Image analysis

Images of the slices were acquired using a computer setup equipped with an Olympus CX31 light microscope (Olympus Corporation, Japan, 10x objective), VideoZavr Scan 2.4 software package (VideoZavr, Russia), and camera VideoZavr Standart VZ-18C23-B (VideoZavr, Russia). The image processing and all measurements were processed using free Fiji ImageJ software (Schindelin et al., 2012). FOS+ cells were manually marked by dots on their images and counted in each of the regions of interest (ROIs) (see below). NeuN+ neurons were defined in ROIs with automatic methods for object segmentation based on the statistical dominance algorithm (SDA) using the SDA plugin for Fiji ImageJ (Figures 1D, E; Nurzynska et al., 2017). To characterize the patterns of the FOS+ cell distribution, heat maps (Figures 1D, E) were created for every slice using kernel density estimation (KDE), a statistical method used for analyzing the spatial distribution of point data (Yuan et al., 2019), using the Matplotlib library for Python 3.10.9.

Spinal cord neural networks sufficient for generation of the locomotion are located in the enlargements (Iwahara et al., 1992), which can be defined by the location of the motoneuron pools innervating limb muscles. In rats, most of the motoneuronal pools innervating the hindlimb muscles are situated within segments L2-L5 (Nicolopoulos-Stournaras and Iles, 1983; Watson et al., 2009; Wenger et al., 2016; Figure 1C); we therefore analyzed only the segments. Five slices for *c-fos* labeling and three slices for NeuN labeling per segment of interest were taken for analysis for all animals of the DAT-WT and DAT-KO groups. For the spinal cord, ROIs were defined on the basis of the division scheme of the spinal gray matter into functional regions presented in our previous study (Merkulyeva et al., 2018) but simplified and modified for the rat spinal cord. According to this scheme, the gray matter of the spinal cord is divided into dorsomedial (DM, medial half of laminae I-IV), dorsolateral (DL, lateral half of laminae I-IV), centromedial (CM, medial half of laminae V-VI, and partially VII), centrolateral (CL, lateral half of laminae V-VI, and partially VII), and ventral zones (V, laminae VIII, IX and major part of lamina VII) (Figure 1D). Since ventral horns have a small number of FOS+ cells, more detailed division of this zone was not carried out.

For the brainstem, three slices from the rostrocaudal level AP Bregma (−8.0 to −8.4) containing CnF and PAG were used (Figure 1C) according to the rat brain atlas (Paxinos and Watson, 2007). For delineation of the brain nuclei borders, wet slices were used (Figure 1E). The light area situated ventrolateral to the trochlear nerve and mesencephalic trigeminal tract is well correspondent to the CnF borders in the (Paxinos and Watson, 2007) rat brain atlas [the dorsal most fibrous part just under inferior colliculi was not included (Figure 1E)]. PAG borders are also visible on the wet slices. In rats like in other mammals, PAG is subdivided into several regions or subnuclei, mainly for dorsomedial, dorsolateral, and ventrolateral ones (Beitz, 1985; Mouton and Holstege, 1994; Silva and McNaughton, 2019) but no cytoarchitectonic borders between them are visible. Subnucleus of interest was the ventrolateral part participating in the locomotor functions (Mouton and Holstege, 1994; Silva and McNaughton, 2019). According to the rat brain atlas (Paxinos and Watson, 2007), we supposed ventrolateral PAG (VLPAG) was located within the upper line delineated from the center of the *aquaeductus sylvii* to the point of lateral border of the PAG, which was at the horizontal level of the ventral extremity of the *inferior colliculi*, and the lower line passing from the lower extremity of trochlear nerve and coming toward the *aquaeductus sylvii* parallel to the upper line (Figure 1E). The borders between the gray and white matter are clearly visible on the wet slices. After immunohistochemical visualization, the wet slices were mounted on the glasses and were photographed using the computer setup equipped with a light microscope. Only after this procedure were slices dehydrated and covered by the mounting media. On the resulting images, the gray and white matter of the spinal cord, also as an area of CnF and VLPAG, were manually outlined in Fiji ImageJ software and measured.

### 2.7 Statistical analysis

Data are presented as mean ± SD. For the multiple comparisons, Kruskal–Wallis test (for unpaired comparisons) and Friedman test (for paired comparisons) with Dunn’s multiple comparisons correction were used. For the single comparisons, a two-tailed Mann–Whitney test was used.

## 3 Results

Previously, alterations in the volume of some brain structures were shown for DAT-KO animals (Reinwald et al., 2022). Therefore, we compared some size characteristics of the spinal cord and brainstem of DAT-KO rats to the DAT-WT ones.

### 3.1 General morphometry of the spinal cord

On average, segments L1-L2, L3-L4, and L5-L6 in DAT-WT rats had similar lengths (*p* ≥ 0.9370; Friedman test). In DAT-KO rats, the minimal values were for the L5-L6 segments (*p* = 0.0063; Friedman test). The DAT-KO rats had a bit shorter spinal segments, but inter-group differences were not statistically significant, for all segments (Table 2).

**TABLE 2:** Length of the lumbar spinal segments in DAT-WT and DAT-KO rats (Kruskal–Wallis test used).

	L1-L2	L3-L4	L5-L6
DAT-WT	5.1 ± 0.6	5.0 ± 0.6	4.4 ± 0.9
DAT-KO	4.8 ± 0.7	4.4 ± 0.5	4.0 ± 0.3
*P*-value	> 0.9000	> 0.9000	> 0.9000

**FIGURE 2:**
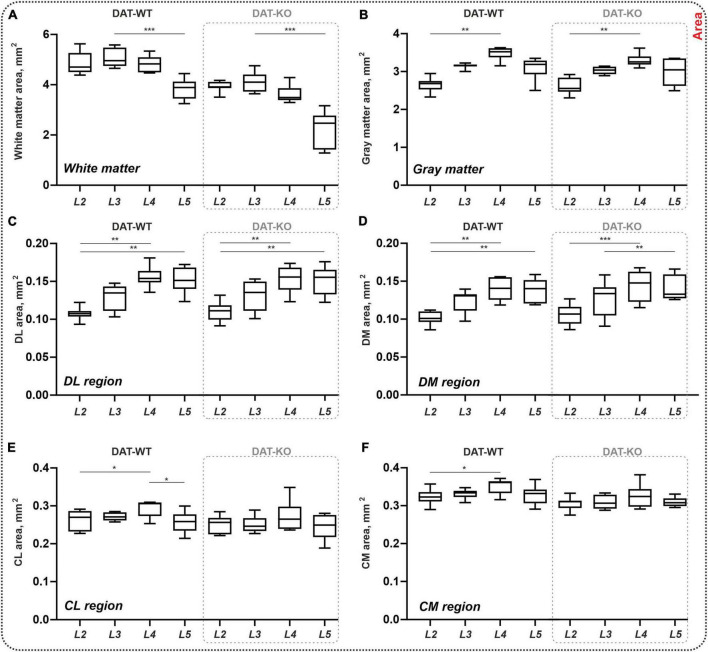
General morphometry of the spinal cord in DAT-WT and DAT-KO rats. **(A,B)** Area of the white and gray matter. **(C–F)** Area of the four regions of interest: DL, DM, CL, CM—dorsolateral, dorsomedial, centrolateral, and centromedial regions. L2-L5—lumbar segments. *p < 0.05; **p < 0.01, ***p < 0.001.

The area of the white and gray matter was also assessed (Figures 2A, B). In both groups, the *white matter* areas were approximately similar for segments L2 and L3, and significantly lower for segment L5 compared to L3 (DAT-WT: *p* = 0.0002, DAT-KO: *p* = 0.0002; Friedman test) (Figure 2A). In parallel, in both groups, the area of the *gray matter* gradually increased from segment L2 to segment L4 (DAT-WT: *p* = 0.0114, DAT-KO: *p* = 0.0056; Friedman test), and thereafter insignificantly decreased from segment L4 to segment L5 (Figure 2B). Despite the trend for the total white matter area being lower in the DAT-KO group (Figure 2A), the difference was not significant (*p* > 0.9999; Kruskal–Wallis test, for all segments).

At the same time, the area of *dorsal regions* of interest increased from L2 to L4-L5 segments in both groups (DAT-WT: *p* = 0.0026 and *p* = 0.0012, DAT-KO: *p* = 0.0029 and *p* = 0.0006, for DL and DM regions; Friedman test) (Figures 2C, D). No inter-group differences were found (*p* > 0.9999; Kruskal–Wallis test, for all segments). In the *central regions* of interest, inter-segmental differences were revealed only in DAT-WT rats: increased area in segment L4 compared to segment L2 (*p* = 0.0427 and *p* = 0.0427, for CL and CM regions; Friedman test), and decreased area in segment L5 compared to segment L4 (*p* = 0.0427, for CL region only; Friedman test) (Figures 2E, F), but not in DAT-KO rats (*p* > 0.9999; Friedman test). No inter-group differences were also observed (*p* > 0.9999; Kruskal–Wallis test, for all segments). Thereby, general morphometric characteristics of the spinal cord are similar in the DAT-WT and DAT-KO rats.

### 3.2 C-fos labeling in the lumbar spinal cord

During the treadmill stepping experiment, the total distances traveled by DAT-WT and DAT-KO rats were significantly different (DAT-WT: 505 ± 17, DAT-KO: 579 ± 6 m; *p* = 0.0286, Mann–Whitney test). It is related to the higher running speed in the DAT-KO rats and corresponds well to hyperlocomotion previously documented for DAT-KO mice and rats (Rossi and Yin, 2015; Reinwald et al., 2022). Despite this, there was no significant difference in the total FOS+ cells in DAT-KO rats vs. DAT-WT rats (207 ± 48 vs. 309 ± 194 cells per segment; *p* = 0.7000). Given this, we used to compare *c-fos* staining: not an absolute number of FOS+ cells but the ratios of FOS+ cells between different ROIs.

The majority of the FOS+ cells were stained in the dorsal horns (laminae II-IV): 66 ± 22% from the total labeling in the DAT-WT group and 66 ± 26% in the DAT-KO group. Less but pronounced labeling was evident within the medial half of the dorsal horns base (laminae V and VI) and within the central gray matter (laminae VII and X); these cells were 29 ± 17 and 29 ± 20% from the total labeling. Solitary labeled cells were also evident in lamina VIII and X. These comprise only 1–11% of the total labeling. No FOS+ cells were revealed within the motoneuronal pools.

**FIGURE 3:**
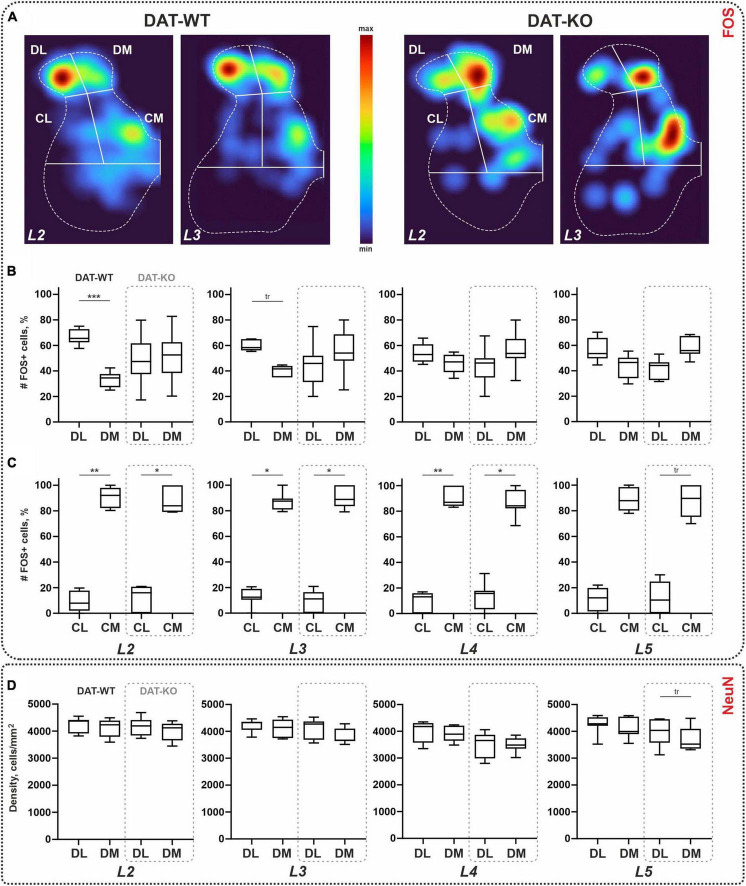
Distribution of the *c-fos*-immunopositive (FOS+) and NeuN-immunopositive cells within the lumbar spinal cord of wild type (DAT-WT) and DAT-knockout (DAT-KO) rats. **(A)** Representative examples of the heat maps of the *c-fos* labeling in L2 and L3 segments of DAT-WT and DAT-KO rats. **(B,C)** A percent number of FOS+ cells in the dorsal **(B)** and intermedial **(C)** regions. **(D)** A density of NeuN+ cells in the dorsal regions. DL, DM, CL, CM—dorsolateral, dorsomedial, centrolateral, and centromedial regions of interest. L2-L5—lumbar segments. *p < 0.05; **p < 0.01; ***p < 0.001; tr—*p* ≤ 0.09.

Within the *dorsal horns*, the ratio of FOS+ cells between the lateral and medial parts of the II-IV laminae was calculated. As can be seen in Figure 3A, in the DAT-WT group, labeled cells mostly occupy the lateral region. A two-fold difference between two regions was statistically significant for segment L2, and an insignificant trend was seen for segment L3 (*p* = 0.0006, *p* = 0.0902; Friedman test) (Figure 3B). In contrast, in the DAT-KO group, either both regions were equally labeled or a trend for the medial region to be more activated was revealed (Figure 3B). The difference between the two regions in these animals was not statistically significant, for all segments (*p* > 0.9999; Friedman test). No inter-group differences in the percent ratio of FOS+ cells were revealed (*p* > 0.9999; Kruskal–Wallis test, for all segments).

In the *central regions*, regardless of group, many more cells were obtained in the medial region (Figure 3C). In general, the ratio between the CL and CM within the group was 1:9. The difference between lateral and medial regions was statistically significant for all segments (*p* = 0.0156, *p* = 0.0089, *p* = 0.008, and *p* = 0.0156; Friedman test) in DAT-WT group, and for segments L2-L4 (*p* = 0.0156, *p* = 0.0201, and *p* = 0.0435; Friedman test) in DAT-KO group (Figure 3C). No inter-group differences in the percent ratio of FOS+ cells were revealed (*p* > 0.9999; Kruskal–Wallis test, for all segments).

In the unstepped rat, only solitary FOS+ cells located within the dorsal horns were observed (one to three per slice), as was previously documented for the unstepped rats (Ahn et al., 2006) and cats (Merkulyeva et al., 2018).

### 3.3 General neuronal population of the dorsal laminae of the spinal cord

One possible reason for the difference between the DAT-WT and DAT-KO groups revealed within the dorsal regions is the total neuronal density in these regions. To test that, we used a NeuN antibody as a marker for the general neuronal population (Mullen et al., 1992); NeuN+ cells were counted within the DL and DM regions for all animals of the DAT-WT and DAT-KO groups.

Averaged values of the cellular density ranged between 3,916–4,259 cell/mm^2^ and 3,513–4,177 cell/mm^2^, in the DL and DM regions (Figure 3D). No differences between DL and DM regions were obtained in the DAT-WT group (*p* > 0.9999; Friedman test), for all segments, and in the DAT-KO group (*p* > 0.3003; Friedman test) in L2-L4 segments (Figure 3D). For segment L5, an insignificant trend for the DL region to be larger compared to the DM region was obtained in the DAT-KO group (*p* = 0.0616; Friedman test) (Figure 3D). No inter-group differences were revealed (*p* > 0.9999; Kruskal–Wallis test, for all segments). Therefore, the difference in the percent number of FOS+ *cells* within segments L2-L3 obtained above is not related to the density of the general neuronal population of the dorsal regions.

We also assessed the percent ratio between the FOS+ cells and the NeuN+ cells. In both regions, it was similar in two regions and two groups (DL region: 4.0–7.5%, in segments L2-L5 of DAT-WT rats, and 3.8–5.5%, in segments L2-L5 of DAT-KO rats; *p* > 0.9999; Kruskal–Wallis test; DM region: 4.5–9.5%, in segments L2-L5 of DAT-WT rats, and 2.8–5.5%, in segments L2-L5 of DAT-KO rats; *p* > 0.9999; Kruskal–Wallis test).

### 3.4 General morphometry of the brainstem

**FIGURE 4:**
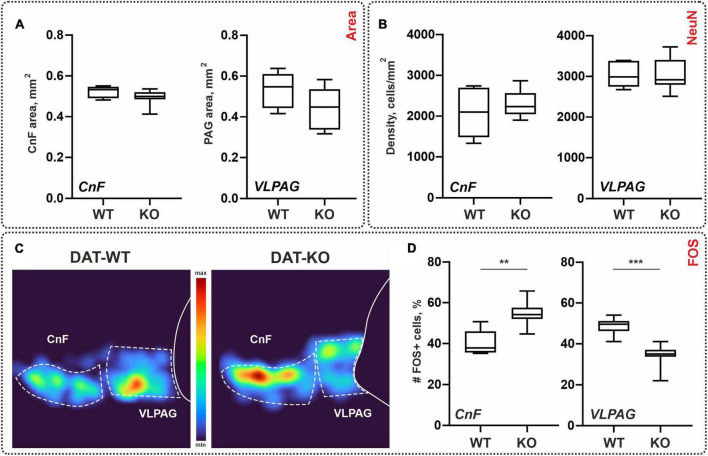
General morphometry and the distribution of the NeuN-immunopositive (NeuN+) and *c-fos*-immunopositive (FOS+) cells within the brainstem nuclei of wild type (DAT-WT) and DAT-knockout (DAT-KO) rats. **(A)** Area of the cuneiform nucleus (CnF) and ventrolateral periaqueductal gray (VLPAG). **(B)** NeuN+ cells in the CnF and VLPAG. **(C)** Representative examples of the heat maps of the distribution of FOS+ cells in DAT-WT and DAT-KO rats. **(D)** A percent number of FOS+ cells in the CnF and VLPAG. **p < 0.01; ***p < 0.001.

Areas of the CnF and VLPAG nuclei ranged between 0.49–0.52 mm^2^ and 0.44–0.53 mm^2^ for DAT-WT and DAT-KO, respectively. A trend for both nuclei of the DAT-KO rats to have lower areas compared with DAT-WT rats (Figure 4A) was obtained; these differences are insignificant (*p* = 0.2284 and *p* = 0.1419; Mann–Whitney test). Therefore, general morphometric characteristics of the brainstem nuclei CnF and VLPAG were similar in the DAT-WT and DAT-KO rats.

### 3.5 General neuronal population of the brainstem

As for the spinal cord, we compared the general neuronal density of the brainstem regions of interest using NeuN antibodies: CnF and VLPAG. The average cellular density of the CnF in the DAT-WT and DAT-KO groups was 2,083 ± 612 cell/mm^2^ and 2,306 ± 319 cell/mm^2^. The average cellular density of the VLPAG in the DAT-WT and DAT-KO groups was 3,033 ± 313 cell/mm^2^ and 3,034 ± 400 cell/mm^2^ (Figure 4B). No inter-group differences were detected (*p* > 0.9999; Friedman test).

For the spinal cord, we also assessed the percent ratio between the FOS+ cells and the NeuN+ cells. In the CnF nucleus, it was similar in two groups (11.4 ± 2.0%, in DAT-WT rats, and 8.8 ± 4.5%, in DAT-KO rats; *p* = 0.3450; Kruskal–Wallis test). In the VLPAG nucleus, it was significantly larger in DAT-WT rats (10.7 ± 1.5%, in DAT-WT rats, and 5.6 ± 3.8%, in DAT-KO rats; *p* = 0.0200; Kruskal–Wallis test). Interestingly, the percentage of number of FOS+ cells in relation to the general neuronal population is similar in the spinal cord and in the brainstem nuclei.

### 3.6 C-fos labeling in the brainstem

Spinal locomotor networks are under supraspinal modulatory influence, and the second task was to compare *c-fos* labeling in the two brainstem nuclei: CnF and VLPAG. Dark stained FOS+ cells were revealed in both the CnF and the VLPAG where they were organized into clear visible clusters (Figure 4C). Previously FOS+ cells within the CnF and VLPAG were also documented after the locomotor task (Opris et al., 2019).

Like for the *c-fos* staining in the spinal cord, we compared not an absolute number but percent of the FOS+ cells assessed relative to the total amount of FOS+ cells within both nuclei. In the DAT-WT group, CnF and VLPAG contained approximately the same number of FOS+ cells (51 vs. 49%; *p* = 0.5625, Wilcoxon test). In the DAT-KO group, VLPAG contained a significantly smaller number of FOS+ cells compared to the CnF (34 vs. 65%; *p* = 0.0078, Wilcoxon test) (Figure 4D). In general, in the DAT-KO group, significantly more FOS+ cells were obtained in the CnF (*p* = 0.0013; Mann–Whitney test), and significantly fewer FOS+ cells were obtained in the VLPAG (*p* = 0.0007; Mann–Whitney test) (Figure 4D).

## 4 Discussion

### 4.1 Distribution of the c-fos staining in the spinal cord of the DAT-knockout rats

The two main results concerning the *c-fos* staining in the spinal cord of the DAT-KO rats are as follows: (1) in contrast to the DAT-WT rats, no mediolateral gradient in the labeled cells exists in the dorsal laminae, and (2) in the intermediate (central) gray matter, no changes were observed. Since the total neuronal numbers of these regions of interest were unchanged in the DAT-KO rats, we believe that the peculiarities of the *c-fos* staining revealed here are related to the functional changes of the neuronal networks of the spinal cord of the DAT-deficient animals.

Nerve terminals containing dopamine are found in the spinal laminae of several mammals like rats, cats, and monkeys (Blessing and Chalmers, 1979; Commissiong et al., 1979; Skagerberg and Lindvall, 1985; Holstege et al., 1996). Dopamine-containing fibers were mainly located within the dorsal horns, in the intermediolateral nucleus, and around the central canal (Mouchet et al., 1982; Skagerberg et al., 1982; Dietl et al., 1985). As was said above, no alterations in the central gray were revealed. This fact is in line with an absence of dopamine decrease in these areas after a midbrain knife-cut in rats, in contrast to a statistically significant dopamine decrease in the dorsal horns (Mouchet et al., 1982). It is possible that spinal generatory networks located within the central gray matter are mainly unaltered in the DAT-deficient rats.

In rats, dopamine-containing fibers are mainly located in the lateral aspects of laminae II-IV, and medial part of lamina VI (Dietl et al., 1985). In cats, the highest dopamine concentration is also detected in the medial part of this lamina (Fleetwood-Walker and Coote, 1981). It was proposed that increased dopamine tone in the DAT-KO animals is the main source for their hyperlocomotion. In this case, the disappearance of the difference between the lateral and medial aspects of the dorsal laminae in the DAT-KO rats may be related to the altered innervation of these regions by the dopamine-containing fibers.

The altered pattern of the *c-fos* staining within the dorsal horns is possibly related to the characteristics of the sensorimotor gating that is mainly realized by the dorsal spinal networks. Previously, the modulatory effects of dopamine on spinal reflexes and as a sequence on the motor output were revealed (Carp and Anderson, 1982; Gajendiran et al., 1996). Dopamine action on the CPGs is either potentiative or suppressive depending upon the dopamine receptor subtypes (Clemens and Hochman, 2004; Humphreys and Whelan, 2012). In the tadpole model, Clemens and Hochman (2004) revealed that a high dopamine concentration preferably recruits a lower-affinity D1 receptor pathway that in turn activates the spinal locomotor CPGs; it corresponds well with the hyperlocomotion obtained in DAT-KO mice and rats. Study of the DAT-KO mice lead to supposition that D1 receptors activation is critical for the expression of repetitive perseverative movements, whereas D2 receptors are necessary for the dopaminergic modulation of the prepulse inhibition that mirrors the sensorimotor gating (Ralph et al., 2001). In rats, the higher level of the D1 receptors was revealed in the ventral horns and the higher level of the D2 receptors in *substantia gelatinosa* (Dubois et al., 1986) or in laminae III-IV and the intermediolateral nucleus (van Dijken et al., 1996). In mice, both types of receptors can be seen in gray matter in figures presented in Zhu et al. (2007). Interestingly, the differences between the lateral and medial aspects of the dorsal horns in the number of neurons containing D1 and D2 receptors can be seen in the figures presented by van Dijken et al. (1996) and Zhu et al. (2007). Therefore, our *c-fos* data for the spinal cord are possibly related to the altered sensorimotor gating in DAT-KO rats.

### 4.2 Distribution of the c-fos staining in the brainstem of the DAT-knockout rats

The two main findings are as follows: (1) DAT-KO rats have more FOS+ cells in the CnF nucleus and (2) fewer FOS+ cells in the VLPAG than DAT-WT rats. As with the spinal cord, no differences in the general neuronal populations of these nuclei were revealed, and we therefore believe that the peculiarities of the *c-fos* staining revealed here are related to the functional changes of the neuronal networks of the spinal cord of the DAT-deficient animals.

The CnF is a part of the mesencephalic locomotor area (Shik and Orlovsky, 1976; Garcia-Rill et al., 1983); its main function is a tonic driving of the spinal locomotor networks by mean of the indirect projections passing to the reticular formation and thereafter to the cervical and lumbar enlargements of the spinal cord (Garcia-Rill et al., 1983; Noga et al., 2003). Electrical stimulation of the MLR is well used for evoking the treadmill locomotion (Shik and Orlovsky, 1976; Noga et al., 1995). We should keep in mind that according to Sinnamon (1993), three main types of locomotion exist: exploratory, appetitive, and defensive; and MLR serves as an integratory center for all of them. Exploratory locomotion is “directed to distal stimuli”; the main function of the defensive locomotion is “to increase the distance between the organism and threatening or painful stimuli” (Sinnamon, 1993). It is obvious that the treadmill locomotion of the intact animal is not as simple as can be supposed, and it does include a defensive component.

Defensive reactions include escape movements, freezing, and confrontational behaviors; one of the key structures for defensive behavior is a PAG (Depaulis et al., 1992), and its different parts (so-called columns) are responsible for different components of the defensive behavior (Bandler and Depaulis, 1991; Sandner et al., 1992). The local chemical activation of the VLPAG leads to the locomotion suppression or immobility (“freezing”) in both rats and cats (Bandler and Depaulis, 1991; Morgan and Carrive, 2001). More FOS+ cells were also documented within the VLPAG after freezing in conditioned rats allowing to propose that this region acts as an integrating center mediating behavioral inhibition (Carrive et al., 1997).

Both the MLR and the VLPAG are under the dopaminergic influence (Beckstead et al., 1979; Gerfen et al., 1982; Rolland et al., 2009; Ryczko et al., 2016; Sharma et al., 2018). Therefore, some alterations in their activity in hyperdopaminergic animals were expected. In the WT rats, the number of the FOS+ cells within the CnF and VLPAG is balanced but not in the DAT-KO group. The higher relative number of the FOS+ cells in the CnF and the lower relative number in the VLPAG in the DAT-KO rats can be interpreted as a higher rate of MLR activity and a lower rate of the VLPAG activity in hyperdopaminergic animals. Our data correspond well with the hyperlocomotion observed in DAT-KO animals under several environmental conditions (Giros et al., 1996; Spielewoy et al., 2000; Rossi and Yin, 2015; Leo et al., 2018; Reinwald et al., 2022) and to the decreased ability of DAT-KO rats to defensive reactions like freezing (Adinolfi et al., 2019).

### 4.3 Generatory and sensorimotor circuits for locomotion

One of general PAG’s functions related to defensive behavior is the regulation of the sensory transmission within the dorsal horns of the spinal cord (Koutsikou et al., 2017); thus, PAG is a specific controller for the spinal sensorimotor circuits. According to one hypothesis, spinal locomotor CPGs consist of two levels: the first one, the “rhythmogenic level,” is composed of pacemaker neurons; the second one, the “pattern formation level,” is composed of multiple interneuronal populations related to the sensory processing (McCrea and Rybak, 2008). According to our data, the rhythmogenic level of the CPGs (located in the central gray matter) is mainly unaffected in the DAT-KO group, but its main source for tonic drive, MLR, is more active compared to the wild type animals. In contrast, the “pattern formation” level of the spinal CPGs seems to be altered in animals lacking DAT; moreover, the controller of these neuronal networks, PAG, is less active compared to the wild type animals. Thereby, key spinal and brainstem structures regulating the locomotor behavior are unbalanced in hyperdopaminergic rats.

### 4.4 General morphometry of the spinal cord and brainstem structures

Previously, a decrease in the volume of *corpus striatum* was obtained in the DAT-KO rats (Reinwald et al., 2022). Comparing the size of the white and gray matter of the spinal cord and the sizes of CnF and VLPAG nuclei did not reveal any valid changes in the DAT-KO rats. Some insignificant trends in the areas can possibly be attributed to the lower body weight of the knockout animals; previously, a link between the body weight and the total brain (Atchley, 1984; Yang et al., 2018) and spinal cord (Fontana et al., 2009) size was documented for rats.

### 4.5 Limitations

It was shown recently that dopamine neurons in the VLPAG differentially regulate pain-related behaviors in male and female mice (Yu et al., 2021). It is possible that the PAG’s neuronal networks responsible for the locomotion also can be unequal in males and females, but only male rats were used in the present study. Another limitation is the PAG is also an important neural substrate for autonomic regulation (Li and Mitchell, 2000); consequently, a decreased number of the FOS+ cells in the VLPAG of the DAT-KO rats can also be related to the altered autonomic regulation, but this point was out of scope for the present paper.

## Data availability statement

The original contributions presented in this study are included in this article/supplementary materials, further inquiries can be directed to the corresponding author.

## Ethics statement

The animal study was approved by the Ethical Committee in the field of Animal Research of Saint Petersburg State University. The study was conducted in accordance with the local legislation and institutional requirements.

## Author contributions

AV: Formal analysis, Investigation, Methodology, Software, Writing – original draft, Writing – review and editing. PS: Methodology, Writing – review and editing. AM: Formal analysis, Investigation, Methodology, Software, Writing – review and editing. ZF: Writing – review and editing. EE: Writing – review and editing. RG: Resources, Writing – review and editing. NM: Conceptualization, Data curation, Formal analysis, Funding acquisition, Investigation, Project administration, Resources, Supervision, Validation, Visualization, Writing – original draft.
